# REVISITING THE ROLE OF PERCEIVED TIME HORIZONS IN SOCIOEMOTIONAL SELECTIVITY THEORY

**DOI:** 10.1093/geroni/igac059.750

**Published:** 2022-12-20

**Authors:** Li Chu, Laura Carstensen

## Abstract

This symposium will consider the ways that time horizons may influence motivation and emotional well-being. Socioemotional selectivity theory postulates that goals and motivation shift from ones about learning and exploration to ones about emotional meaning as time horizons grow constrained as people age. This theory maintains that a focus on emotional goals explains why older adults tend to show better emotional well-being compared to younger adults. Many studies use the Future Time Perspective scale developed by Carstensen and Lang (1996) to measure perceived time left in life. However, several studies find more limited future time perspective does not contribute to better emotional well-being nor more positivity bias in cognitive processing and in some cases predicts poorer emotional well-being. The first presentation will focus on the future time perspective scale and its underlying structure. The second presentation will discuss the association between future time perspective and psychosocial well-being during the COVID-19 pandemic. The third presentation will review different measures of time perception and examine the relationship between future time perspective on age-related positivity effect. The last presentation will introduce a new concept and measurement, time savoring. Laura Carstensen will synthesize findings and offer insights regarding future life-span research on motivation and well-being.

